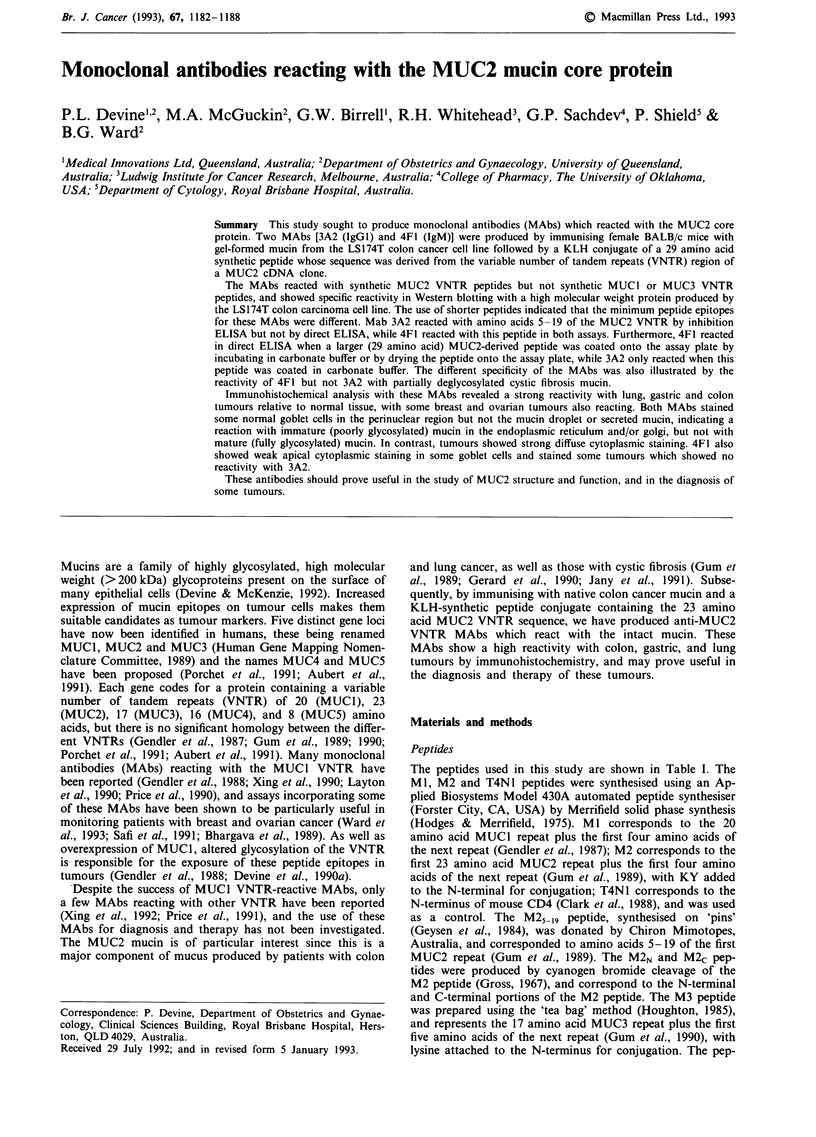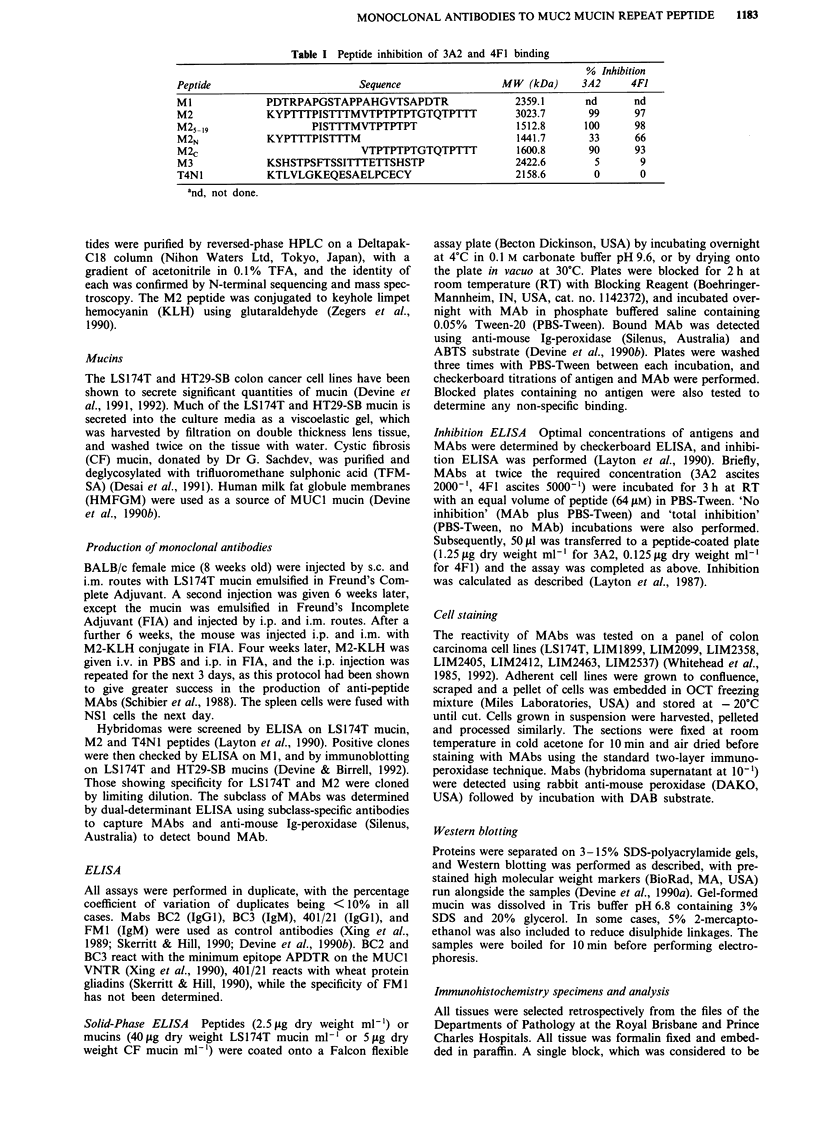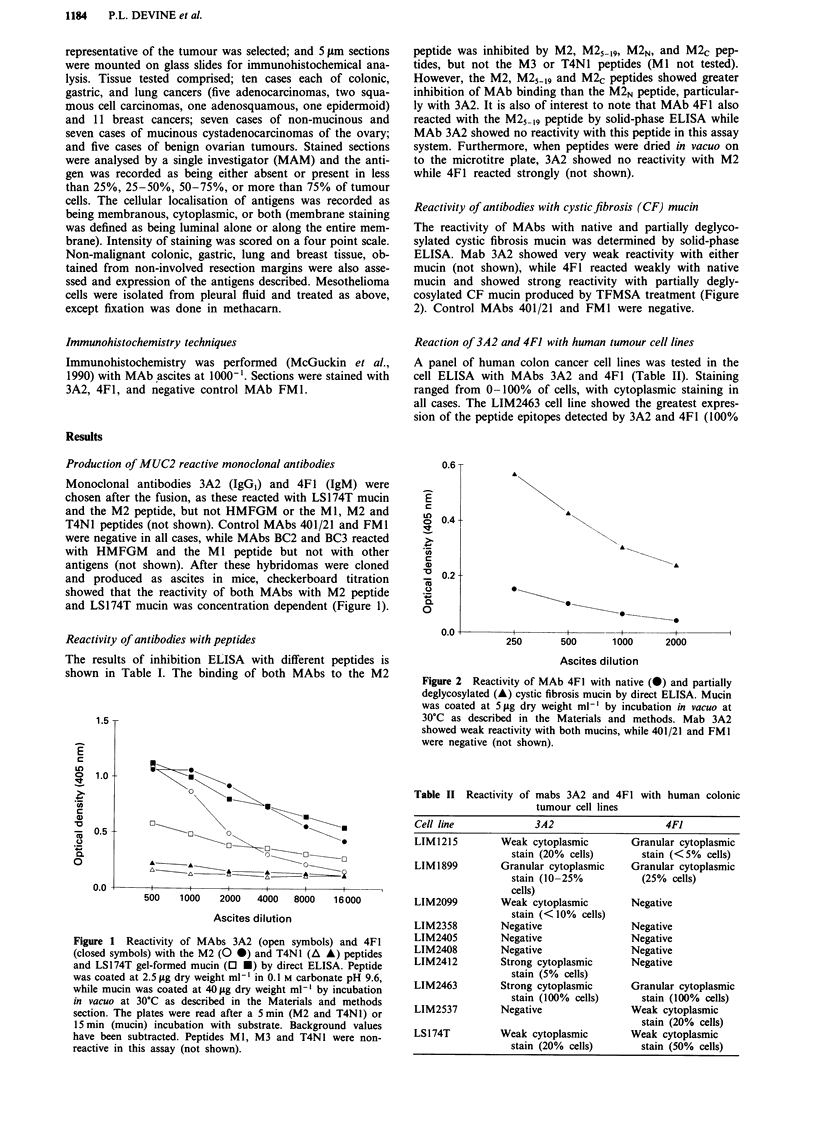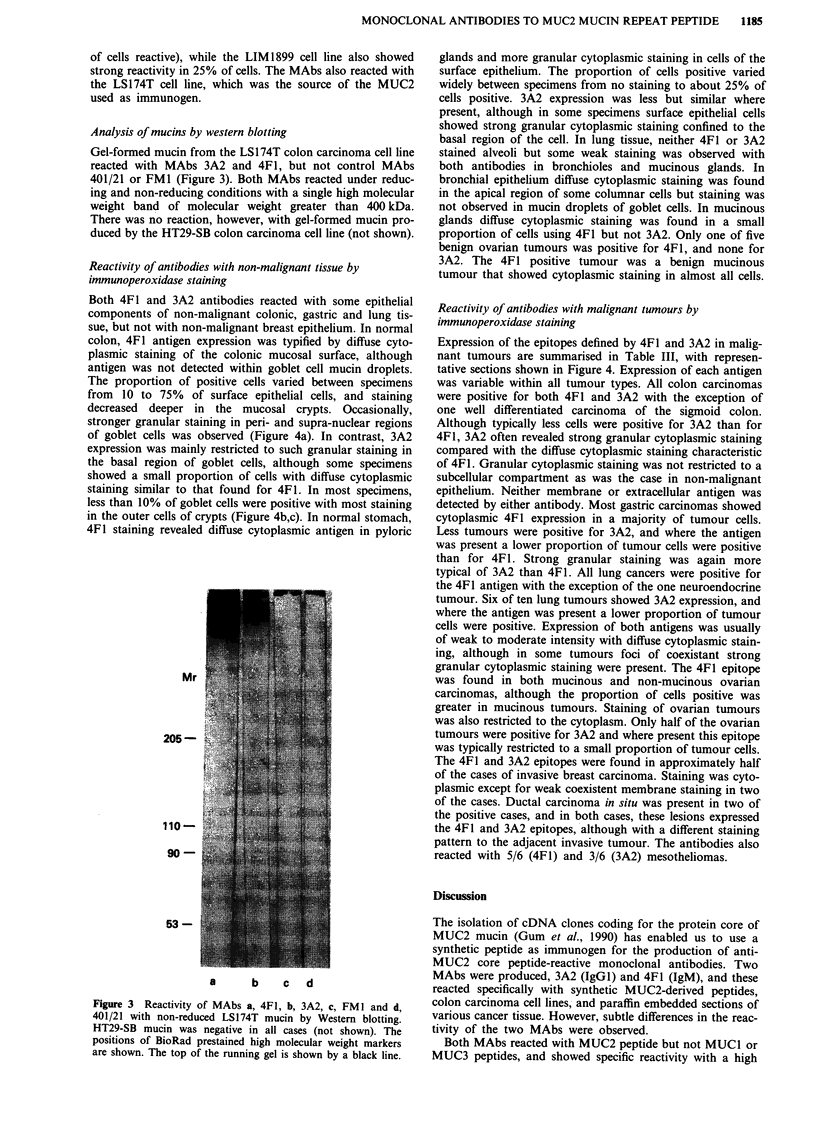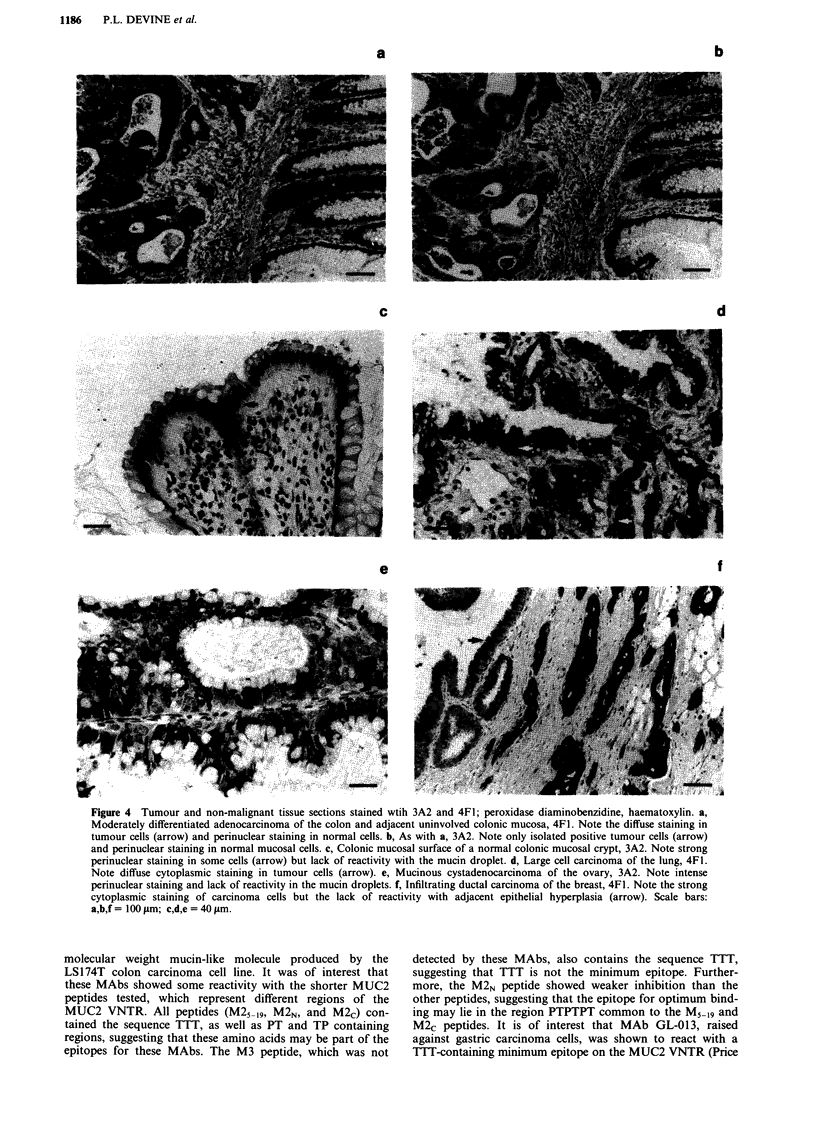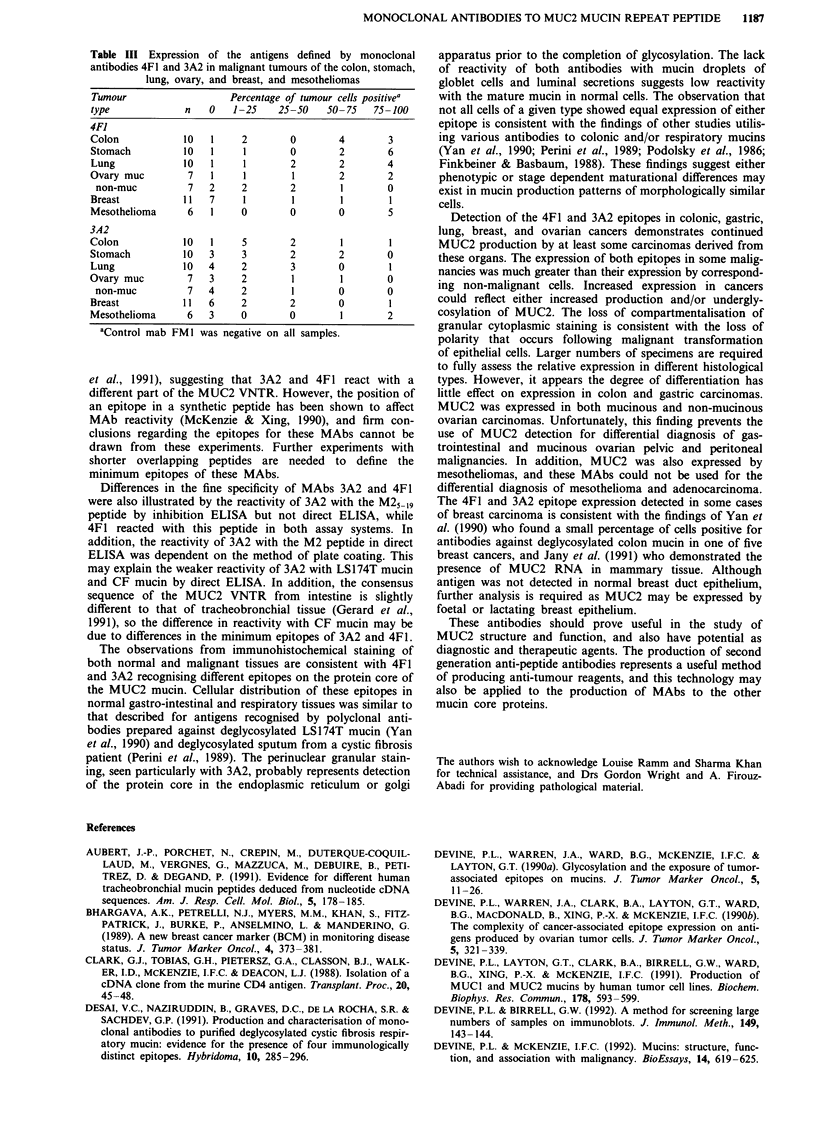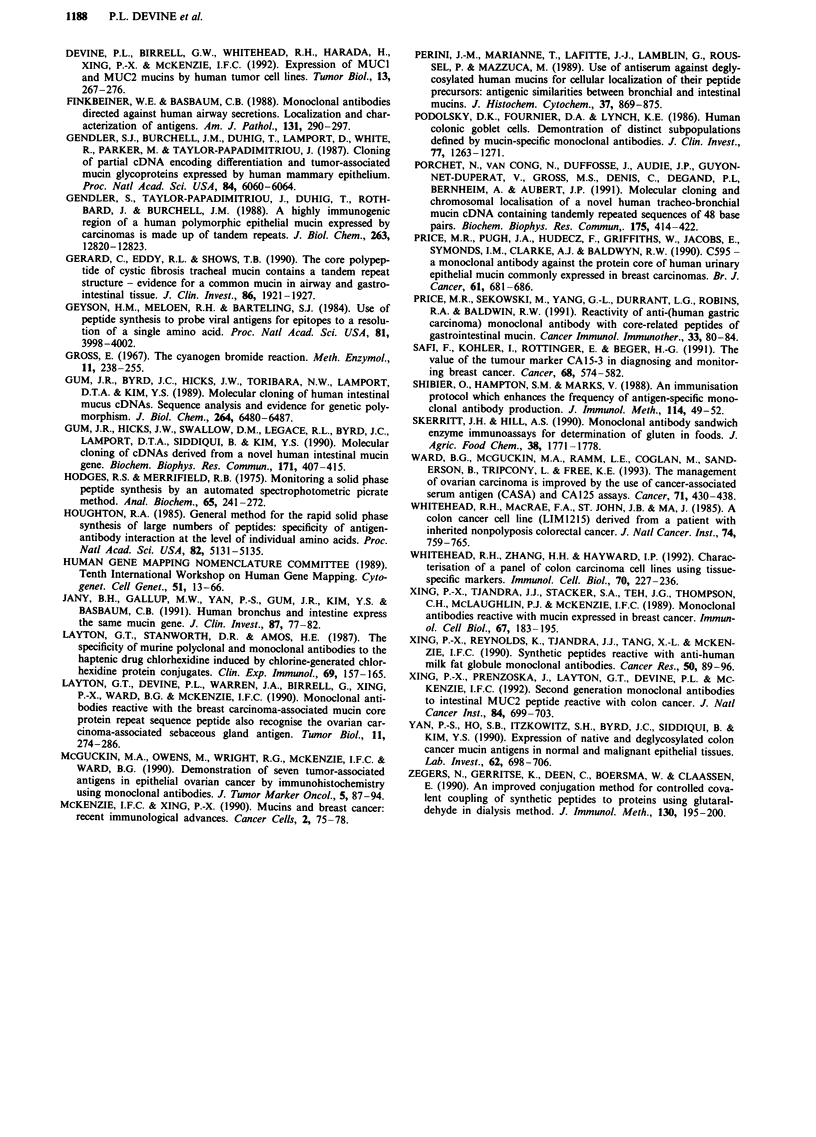# Monoclonal antibodies reacting with the MUC2 mucin core protein.

**DOI:** 10.1038/bjc.1993.223

**Published:** 1993-06

**Authors:** P. L. Devine, M. A. McGuckin, G. W. Birrell, R. H. Whitehead, G. P. Sachdev, P. Shield, B. G. Ward

**Affiliations:** Medical Innovations Ltd, Queensland, Australia.

## Abstract

**Images:**


Br. J. Cancer (1993), 67, 1182-1188 © Macmillan Press Ltd., 1993

### Monoclonal antibodies reacting with the MUC2 mucin core protein

P.L. Devine[1,2], M.A. McGuckin[2], G.W. Birrell[1], R.H. Whitehead[3], G.P. Sachdev[4], P. Shield[5] & B.G. Ward[2]

[1]*Medical Innovations Ltd, Queensland, Australia;* [2]*Department of Obstetrics and Gynaecology, University of Queensland, Australia;* [3]*Ludwig Institute for Cancer Research, Melbourne, Australia;* [4]*College of Pharmacy, The University of Oklahoma, USA;* [5]*Department of Cytology, Royal Brisbane Hospital, Australia.*

**Summary** This study sought to produce monoclonal antibodies (MAbs) which reacted with the MUC2 core protein. Two MAbs [3A2 (IgG1) and 4F1 (IgM)] were produced by immunising female BALB/c mice with gel-formed mucin from the LS174T colon cancer cell line followed by a KLH conjugate of a 29 amino acid synthetic peptide whose sequence was derived from the variable number of tandem repeats (VNTR) region of a MUC2 cDNA clone.

The MAbs reacted with synthetic MUC2 VNTR peptides but not synthetic MUC1 or MUC3 VNTR peptides, and showed specific reactivity in Western blotting with a high molecular weight protein produced by the LS174T colon carcinoma cell line. The use of shorter peptides indicated that the minimum peptide epitopes for these MAbs were different. Mab 3A2 reacted with amino acids 5-19 of the MUC2 VNTR by inhibition ELISA but not by direct ELISA, while 4F1 reacted with this peptide in both assays. Furthermore, 4F1 reacted in direct ELISA when a larger (29 amino acid) MUC2-derived peptide was coated onto the assay plate by incubating in carbonate buffer or by drying the peptide onto the assay plate, while 3A2 only reacted when this peptide was coated in carbonate buffer. The different specificity of the MAbs was also illustrated by the reactivity of 4F1 but not 3A2 with partially deglycosylated cystic fibrosis mucin.

Immunohistochemical analysis with these MAbs revealed a strong reactivity with lung, gastric and colon tumours relative to normal tissue, with some breast and ovarian tumours also reacting. Both MAbs stained some normal goblet cells in the perinuclear region but not the mucin droplet or secreted mucin, indicating a reaction with immature (poorly glycosylated) mucin in the endoplasmic reticulum and/or golgi, but not with mature (fully glycosylated) mucin. In contrast, tumours showed strong diffuse cytoplasmic staining. 4F1 also showed weak apical cytoplasmic staining in some goblet cells and stained some tumours which showed no reactivity with 3A2.

These antibodies should prove useful in the study of MUC2 structure and function, and in the diagnosis of some tumours.

Mucins are a family of highly glycosylated, high molecular weight (>200 kDa) glycoproteins present on the surface of many epithelial cells (Devine & McKenzie, 1992). Increased expression of mucin epitopes on tumour cells makes them suitable candidates as tumour markers. Five distinct gene loci have now been identified in humans, these being renamed MUC1, MUC2 and MUC3 (Human Gene Mapping Nomenclature Committee, 1989) and the names MUC4 and MUC5 have been proposed (Porchet *et al.*, 1991; Aubert *et al.*, 1991). Each gene codes for a protein containing a variable number of tandem repeats (VNTR) of 20 (MUC1), 23 (MUC2), 17 (MUC3), 16 (MUC4), and 8 (MUC5) amino acids, but there is no significant homology between the different VNTRs (Gendler *et al.*, 1987; Gum *et al.*, 1989; 1990; Porchet *et al.*, 1991; Aubert *et al.*, 1991). Many monoclonal antibodies (MAbs) reacting with the MUC1 VNTR have been reported (Gendler *et al.*, 1988; Xing *et al.*, 1990; Layton *et al.*, 1990; Price *et al.*, 1990), and assays incorporating some of these MAbs have been shown to be particularly useful in monitoring patients with breast and ovarian cancer (Ward *et al.*, 1993; Safi *et al.*, 1991; Bhargava *et al.*, 1989). As well as overexpression of MUC1, altered glycosylation of the VNTR is responsible for the exposure of these peptide epitopes in tumours (Gendler *et al.*, 1988; Devine *et al.*, 1990*a*).

Despite the success of MUC1 VNTR-reactive MAbs, only a few MAbs reacting with other VNTR have been reported (Xing *et al.*, 1992; Price *et al.*, 1991), and the use of these MAbs for diagnosis and therapy has not been investigated. The MUC2 mucin is of particular interest since this is a major component of mucus produced by patients with colon and lung cancer, as well as those with cystic fibrosis (Gum *et al.*, 1989; Gerard *et al.*, 1990; Jany *et al.*, 1991). Subsequently, by immunising with native colon cancer mucin and a KLH-synthetic peptide conjugate containing the 23 amino acid MUC2 VNTR sequence, we have produced anti-MUC2 VNTR MAbs which react with the intact mucin. These MAbs show a high reactivity with colon, gastric, and lung tumours by immunohistochemistry, and may prove useful in the diagnosis and therapy of these tumours.

Correspondence: P. Devine, Department of Obstetrics and Gynaecology, Clinical Sciences Building, Royal Brisbane Hospital, Herston, QLD 4029, Australia.
Received 29 July 1992; and in revised form 5 January 1993.

### Materials and methods

### *Peptides*

The peptides used in this study are shown in Table I. The M1, M2 and T4N1 peptides were synthesised using an Applied Biosystems Model 430A automated peptide synthesiser (Forster City, CA, USA) by Merrifield solid phase synthesis (Hodges & Merrifield, 1975). M1 corresponds to the 20 amino acid MUC1 repeat plus the first four amino acids of the next repeat (Gendler *et al.*, 1987); M2 corresponds to the first 23 amino acid MUC2 repeat plus the first four amino acids of the next repeat (Gum *et al.*, 1989), with KY added to the N-terminal for conjugation; T4N1 corresponds to the N-terminus of mouse CD4 (Clark *et al.*, 1988), and was used as a control. The $M2_{5-19}$ peptide, synthesised on 'pins' (Geysen *et al.*, 1984), was donated by Chiron Mimotopes, Australia, and corresponded to amino acids 5-19 of the first MUC2 repeat (Gum *et al.*, 1989). The $M2_N$ and $M2_C$ peptides were produced by cyanogen bromide cleavage of the M2 peptide (Gross, 1967), and correspond to the N-terminal and C-terminal portions of the M2 peptide. The M3 peptide was prepared using the 'tea bag' method (Houghton, 1985), and represents the 17 amino acid MUC3 repeat plus the first five amino acids of the next repeat (Gum *et al.*, 1990), with lysine attached to the N-terminus for conjugation. The pep-

**Table I** Peptide inhibition of 3A2 and 4F1 binding

| *Peptide* | *Sequence* | *MW (kDa)* | *% Inhibition* 3A2 | *% Inhibition* 4F1 |
|---|---|---|---|---|
| M1 | PDTRPAPGSTAPPAHGVTSAPDTR | 2359.1 | nd | nd |
| M2 | KYPTTTPISTTTMVTPTPTPTGTQTPTTT | 3023.7 | 99 | 97 |
| $M2_{5-19}$ | PISTTTMVTPTPTPT | 1512.8 | 100 | 98 |
| $M2_N$ | KYPTTTPISTTTM | 1441.7 | 33 | 66 |
| $M2_C$ | VTPTPTPTGTQTPTTT | 1600.8 | 90 | 93 |
| M3 | KSHSTPSFTSSITTTETTSHSTP | 2422.6 | 5 | 9 |
| T4N1 | KTLVLGKEQESAELPCECY | 2158.6 | 0 | 0 |

[a]nd, not done.

tides were purified by reversed-phase HPLC on a Deltapak-C18 column (Nihon Waters Ltd, Tokyo, Japan), with a gradient of acetonitrile in 0.1% TFA, and the identity of each was confirmed by N-terminal sequencing and mass spectroscopy. The M2 peptide was conjugated to keyhole limpet hemocyanin (KLH) using glutaraldehyde (Zegers *et al.*, 1990).

*Mucins*

The LS174T and HT29-SB colon cancer cell lines have been shown to secrete significant quantities of mucin (Devine *et al.*, 1991, 1992). Much of the LS174T and HT29-SB mucin is secreted into the culture media as a viscoelastic gel, which was harvested by filtration on double thickness lens tissue, and washed twice on the tissue with water. Cystic fibrosis (CF) mucin, donated by Dr G. Sachdev, was purified and deglycosylated with trifluoromethane sulphonic acid (TFMSA) (Desai *et al.*, 1991). Human milk fat globule membranes (HMFGM) were used as a source of MUC1 mucin (Devine *et al.*, 1990*b*).

*Production of monoclonal antibodies*

BALB/c female mice (8 weeks old) were injected by s.c. and i.m. routes with LS174T mucin emulsified in Freund's Complete Adjuvant. A second injection was given 6 weeks later, except the mucin was emulsified in Freund's Incomplete Adjuvant (FIA) and injected by i.p. and i.m. routes. After a further 6 weeks, the mouse was injected i.p. and i.m. with M2-KLH conjugate in FIA. Four weeks later, M2-KLH was given i.v. in PBS and i.p. in FIA, and the i.p. injection was repeated for the next 3 days, as this protocol had been shown to give greater success in the production of anti-peptide MAbs (Schibier *et al.*, 1988). The spleen cells were fused with NS1 cells the next day.

Hybridomas were screened by ELISA on LS174T mucin, M2 and T4N1 peptides (Layton *et al.*, 1990). Positive clones were then checked by ELISA on M1, and by immunoblotting on LS174T and HT29-SB mucins (Devine & Birrell, 1992). Those showing specificity for LS174T and M2 were cloned by limiting dilution. The subclass of MAbs was determined by dual-determinant ELISA using subclass-specific antibodies to capture MAbs and anti-mouse Ig-peroxidase (Silenus, Australia) to detect bound MAb.

*ELISA*

All assays were performed in duplicate, with the percentage coefficient of variation of duplicates being $<10\%$ in all cases. Mabs BC2 (IgG1), BC3 (IgM), 401/21 (IgG1), and FM1 (IgM) were used as control antibodies (Xing *et al.*, 1989; Skerritt & Hill, 1990; Devine *et al.*, 1990*b*). BC2 and BC3 react with the minimum epitope APDTR on the MUC1 VNTR (Xing *et al.*, 1990), 401/21 reacts with wheat protein gliadins (Skerritt & Hill, 1990), while the specificity of FM1 has not been determined.

*Solid-Phase ELISA* Peptides (2.5 µg dry weight $ml^{-1}$) or mucins (40 µg dry weight LS174T mucin $ml^{-1}$ or 5 µg dry weight CF mucin $ml^{-1}$) were coated onto a Falcon flexible assay plate (Becton Dickinson, USA) by incubating overnight at 4°C in 0.1 M carbonate buffer pH 9.6, or by drying onto the plate *in vacuo* at 30°C. Plates were blocked for 2 h at room temperature (RT) with Blocking Reagent (Boehringer-Mannheim, IN, USA, cat. no. 1142372), and incubated overnight with MAb in phosphate buffered saline containing 0.05% Tween-20 (PBS-Tween). Bound MAb was detected using anti-mouse Ig-peroxidase (Silenus, Australia) and ABTS substrate (Devine *et al.*, 1990*b*). Plates were washed three times with PBS-Tween between each incubation, and checkerboard titrations of antigen and MAb were performed. Blocked plates containing no antigen were also tested to determine any non-specific binding.

*Inhibition ELISA* Optimal concentrations of antigens and MAbs were determined by checkerboard ELISA, and inhibition ELISA was performed (Layton *et al.*, 1990). Briefly, MAbs at twice the required concentration (3A2 ascites $2000^{-1}$, 4F1 ascites $5000^{-1}$) were incubated for 3 h at RT with an equal volume of peptide (64 µM) in PBS-Tween. 'No inhibition' (MAb plus PBS-Tween) and 'total inhibition' (PBS-Tween, no MAb) incubations were also performed. Subsequently, 50 µl was transferred to a peptide-coated plate (1.25 µg dry weight $ml^{-1}$ for 3A2, 0.125 µg dry weight $ml^{-1}$ for 4F1) and the assay was completed as above. Inhibition was calculated as described (Layton *et al.*, 1987).

*Cell staining*

The reactivity of MAbs was tested on a panel of colon carcinoma cell lines (LS174T, LIM1899, LIM2099, LIM2358, LIM2405, LIM2412, LIM2463, LIM2537) (Whitehead *et al.*, 1985, 1992). Adherent cell lines were grown to confluence, scraped and a pellet of cells was embedded in OCT freezing mixture (Miles Laboratories, USA) and stored at $-20$°C until cut. Cells grown in suspension were harvested, pelleted and processed similarly. The sections were fixed at room temperature in cold acetone for 10 min and air dried before staining with MAbs using the standard two-layer immunoperoxidase technique. Mabs (hybridoma supernatant at $10^{-1}$) were detected using rabbit anti-mouse peroxidase (DAKO, USA) followed by incubation with DAB substrate.

*Western blotting*

Proteins were separated on 3–15% SDS-polyacrylamide gels, and Western blotting was performed as described, with prestained high molecular weight markers (BioRad, MA, USA) run alongside the samples (Devine *et al.*, 1990*a*). Gel-formed mucin was dissolved in Tris buffer pH 6.8 containing 3% SDS and 20% glycerol. In some cases, 5% 2-mercaptoethanol was also included to reduce disulphide linkages. The samples were boiled for 10 min before performing electrophoresis.

*Immunohistochemistry specimens and analysis*

All tissues were selected retrospectively from the files of the Departments of Pathology at the Royal Brisbane and Prince Charles Hospitals. All tissue was formalin fixed and embedded in paraffin. A single block, which was considered to be representative of the tumour was selected; and 5 µm sections were mounted on glass slides for immunohistochemical analysis. Tissue tested comprised; ten cases each of colonic, gastric, and lung cancers (five adenocarcinomas, two squamous cell carcinomas, one adenosquamous, one epidermoid) and 11 breast cancers; seven cases of non-mucinous and seven cases of mucinous cystadenocarcinomas of the ovary; and five cases of benign ovarian tumours. Stained sections were analysed by a single investigator (MAM) and the antigen was recorded as being either absent or present in less than 25%, 25–50%, 50–75%, or more than 75% of tumour cells. The cellular localisation of antigens was recorded as being membranous, cytoplasmic, or both (membrane staining was defined as being luminal alone or along the entire membrane). Intensity of staining was scored on a four point scale. Non-malignant colonic, gastric, lung and breast tissue, obtained from non-involved resection margins were also assessed and expression of the antigens described. Mesothelioma cells were isolated from pleural fluid and treated as above, except fixation was done in methacarn.

*Immunohistochemistry techniques*

Immunohistochemistry was performed (McGuckin *et al.*, 1990) with MAb ascites at $1000^{-1}$. Sections were stained with 3A2, 4F1, and negative control MAb FM1.

### Results

*Production of MUC2 reactive monoclonal antibodies*

Monoclonal antibodies 3A2 ($IgG_1$) and 4F1 (IgM) were chosen after the fusion, as these reacted with LS174T mucin and the M2 peptide, but not HMFGM or the M1, M2 and T4N1 peptides (not shown). Control MAbs 401/21 and FM1 were negative in all cases, while MAbs BC2 and BC3 reacted with HMFGM and the M1 peptide but not with other antigens (not shown). After these hybridomas were cloned and produced as ascites in mice, checkerboard titration showed that the reactivity of both MAbs with M2 peptide and LS174T mucin was concentration dependent (Figure 1).

*Reactivity of antibodies with peptides*

The results of inhibition ELISA with different peptides is shown in Table I. The binding of both MAbs to the M2 peptide was inhibited by M2, $M2_{5-19}$, $M2_N$, and $M2_C$ peptides, but not the M3 or T4N1 peptides (M1 not tested). However, the M2, $M2_{5-19}$ and $M2_C$ peptides showed greater inhibition of MAb binding than the $M2_N$ peptide, particularly with 3A2. It is also of interest to note that MAb 4F1 also reacted with the $M2_{5-19}$ peptide by solid-phase ELISA while MAb 3A2 showed no reactivity with this peptide in this assay system. Furthermore, when peptides were dried *in vacuo* on to the microtitre plate, 3A2 showed no reactivity with M2 while 4F1 reacted strongly (not shown).

**Figure 1** Reactivity of MAbs 3A2 (open symbols) and 4F1 (closed symbols) with the M2 (○ ●) and T4N1 (△ ▲) peptides and LS174T gel-formed mucin (□ ■) by direct ELISA. Peptide was coated at 2.5 µg dry weight $ml^{-1}$ in 0.1 M carbonate pH 9.6, while mucin was coated at 40 µg dry weight $ml^{-1}$ by incubation *in vacuo* at 30°C as described in the Materials and methods section. The plates were read after a 5 min (M2 and T4N1) or 15 min (mucin) incubation with substrate. Background values have been subtracted. Peptides M1, M3 and T4N1 were non-reactive in this assay (not shown).

*Reactivity of antibodies with cystic fibrosis (CF) mucin*

The reactivity of MAbs with native and partially deglycosylated cystic fibrosis mucin was determined by solid-phase ELISA. Mab 3A2 showed very weak reactivity with either mucin (not shown), while 4F1 reacted weakly with native mucin and showed strong reactivity with partially deglycosylated CF mucin produced by TFMSA treatment (Figure 2). Control MAbs 401/21 and FM1 were negative.

*Reaction of 3A2 and 4F1 with human tumour cell lines*

A panel of human colon cancer cell lines was tested in the cell ELISA with MAbs 3A2 and 4F1 (Table II). Staining ranged from 0–100% of cells, with cytoplasmic staining in all cases. The LIM2463 cell line showed the greatest expression of the peptide epitopes detected by 3A2 and 4F1 (100%

**Figure 2** Reactivity of MAb 4F1 with native (●) and partially deglycosylated (▲) cystic fibrosis mucin by direct ELISA. Mucin was coated at 5 µg dry weight $ml^{-1}$ by incubation *in vacuo* at 30°C as described in the Materials and methods. Mab 3A2 showed weak reactivity with both mucins, while 401/21 and FM1 were negative (not shown).

**Table II** Reactivity of mabs 3A2 and 4F1 with human colonic tumour cell lines

| *Cell line* | *3A2* | *4F1* |
|---|---|---|
| LIM1215 | Weak cytoplasmic stain (20% cells) | Granular cytoplasmic stain (<5% cells) |
| LIM1899 | Granular cytoplasmic stain (10–25% cells) | Granular cytoplasmic (25% cells) |
| LIM2099 | Weak cytoplasmic stain (<10% cells) | Negative |
| LIM2358 | Negative | Negative |
| LIM2405 | Negative | Negative |
| LIM2408 | Negative | Negative |
| LIM2412 | Strong cytoplasmic stain (5% cells) | Negative |
| LIM2463 | Strong cytoplasmic stain (100% cells) | Granular cytoplasmic stain (100% cells) |
| LIM2537 | Negative | Weak cytoplasmic stain (20% cells) |
| LS174T | Weak cytoplasmic stain (20% cells) | Weak cytoplasmic stain (50% cells) |

of cells reactive), while the LIM1899 cell line also showed strong reactivity in 25% of cells. The MAbs also reacted with the LS174T cell line, which was the source of the MUC2 used as immunogen.

### *Analysis of mucins by western blotting*

Gel-formed mucin from the LS174T colon carcinoma cell line reacted with MAbs 3A2 and 4F1, but not control MAbs 401/21 or FM1 (Figure 3). Both MAbs reacted under reducing and non-reducing conditions with a single high molecular weight band of molecular weight greater than 400 kDa. There was no reaction, however, with gel-formed mucin produced by the HT29-SB colon carcinoma cell line (not shown).

### *Reactivity of antibodies with non-malignant tissue by immunoperoxidase staining*

Both 4F1 and 3A2 antibodies reacted with some epithelial components of non-malignant colonic, gastric and lung tissue, but not with non-malignant breast epithelium. In normal colon, 4F1 antigen expression was typified by diffuse cytoplasmic staining of the colonic mucosal surface, although antigen was not detected within goblet cell mucin droplets. The proportion of positive cells varied between specimens from 10 to 75% of surface epithelial cells, and staining decreased deeper in the mucosal crypts. Occasionally, stronger granular staining in peri- and supra-nuclear regions of goblet cells was observed (Figure 4a). In contrast, 3A2 expression was mainly restricted to such granular staining in the basal region of goblet cells, although some specimens showed a small proportion of cells with diffuse cytoplasmic staining similar to that found for 4F1. In most specimens, less than 10% of goblet cells were positive with most staining in the outer cells of crypts (Figure 4b,c). In normal stomach, 4F1 staining revealed diffuse cytoplasmic antigen in pyloric glands and more granular cytoplasmic staining in cells of the surface epithelium. The proportion of cells positive varied widely between specimens from no staining to about 25% of cells positive. 3A2 expression was less but similar where present, although in some specimens surface epithelial cells showed strong granular cytoplasmic staining confined to the basal region of the cell. In lung tissue, neither 4F1 or 3A2 stained alveoli but some weak staining was observed with both antibodies in bronchioles and mucinous glands. In bronchial epithelium diffuse cytoplasmic staining was found in the apical region of some columnar cells but staining was not observed in mucin droplets of goblet cells. In mucinous glands diffuse cytoplasmic staining was found in a small proportion of cells using 4F1 but not 3A2. Only one of five benign ovarian tumours was positive for 4F1, and none for 3A2. The 4F1 positive tumour was a benign mucinous tumour that showed cytoplasmic staining in almost all cells.

**Figure 3** Reactivity of MAbs **a**, 4F1, **b**, 3A2, **c**, FM1 and **d**, 401/21 with non-reduced LS174T mucin by Western blotting. HT29-SB mucin was negative in all cases (not shown). The positions of BioRad prestained high molecular weight markers are shown. The top of the running gel is shown by a black line.

### *Reactivity of antibodies with malignant tumours by immunoperoxidase staining*

Expression of the epitopes defined by 4F1 and 3A2 in malignant tumours are summarised in Table III, with representative sections shown in Figure 4. Expression of each antigen was variable within all tumour types. All colon carcinomas were positive for both 4F1 and 3A2 with the exception of one well differentiated carcinoma of the sigmoid colon. Although typically less cells were positive for 3A2 than for 4F1, 3A2 often revealed strong granular cytoplasmic staining compared with the diffuse cytoplasmic staining characteristic of 4F1. Granular cytoplasmic staining was not restricted to a subcellular compartment as was the case in non-malignant epithelium. Neither membrane or extracellular antigen was detected by either antibody. Most gastric carcinomas showed cytoplasmic 4F1 expression in a majority of tumour cells. Less tumours were positive for 3A2, and where the antigen was present a lower proportion of tumour cells were positive than for 4F1. Strong granular staining was again more typical of 3A2 than 4F1. All lung cancers were positive for the 4F1 antigen with the exception of the one neuroendocrine tumour. Six of ten lung tumours showed 3A2 expression, and where the antigen was present a lower proportion of tumour cells were positive. Expression of both antigens was usually of weak to moderate intensity with diffuse cytoplasmic staining, although in some tumours foci of coexistant strong granular cytoplasmic staining were present. The 4F1 epitope was found in both mucinous and non-mucinous ovarian carcinomas, although the proportion of cells positive was greater in mucinous tumours. Staining of ovarian tumours was also restricted to the cytoplasm. Only half of the ovarian tumours were positive for 3A2 and where present this epitope was typically restricted to a small proportion of tumour cells. The 4F1 and 3A2 epitopes were found in approximately half of the cases of invasive breast carcinoma. Staining was cytoplasmic except for weak coexistent membrane staining in two of the cases. Ductal carcinoma *in situ* was present in two of the positive cases, and in both cases, these lesions expressed the 4F1 and 3A2 epitopes, although with a different staining pattern to the adjacent invasive tumour. The antibodies also reacted with 5/6 (4F1) and 3/6 (3A2) mesotheliomas.

### Discussion

The isolation of cDNA clones coding for the protein core of MUC2 mucin (Gum *et al.*, 1990) has enabled us to use a synthetic peptide as immunogen for the production of anti-MUC2 core peptide-reactive monoclonal antibodies. Two MAbs were produced, 3A2 (IgG1) and 4F1 (IgM), and these reacted specifically with synthetic MUC2-derived peptides, colon carcinoma cell lines, and paraffin embedded sections of various cancer tissue. However, subtle differences in the reactivity of the two MAbs were observed.

Both MAbs reacted with MUC2 peptide but not MUC1 or MUC3 peptides, and showed specific reactivity with a high

**Figure 4** Tumour and non-malignant tissue sections stained wtih 3A2 and 4F1; peroxidase diaminobenzidine, haematoxylin. **a**, Moderately differentiated adenocarcinoma of the colon and adjacent uninvolved colonic mucosa, 4F1. Note the diffuse staining in tumour cells (arrow) and perinuclear staining in normal cells. **b**, As with **a**, 3A2. Note only isolated positive tumour cells (arrow) and perinuclear staining in normal mucosal cells. **c**, Colonic mucosal surface of a normal colonic mucosal crypt, 3A2. Note strong perinuclear staining in some cells (arrow) but lack of reactivity with the mucin droplet. **d**, Large cell carcinoma of the lung, 4F1. Note diffuse cytoplasmic staining in tumour cells (arrow). **e**, Mucinous cystadenocarcinoma of the ovary, 3A2. Note intense perinuclear staining and lack of reactivity in the mucin droplets. **f**, Infiltrating ductal carcinoma of the breast, 4F1. Note the strong cytoplasmic staining of carcinoma cells but the lack of reactivity with adjacent epithelial hyperplasia (arrow). Scale bars: **a,b,f** = 100 μm; **c,d,e** = 40 μm.

molecular weight mucin-like molecule produced by the LS174T colon carcinoma cell line. It was of interest that these MAbs showed some reactivity with the shorter MUC2 peptides tested, which represent different regions of the MUC2 VNTR. All peptides ($M2_{5-19}$, $M2_N$, and $M2_C$) contained the sequence TTT, as well as PT and TP containing regions, suggesting that these amino acids may be part of the epitopes for these MAbs. The M3 peptide, which was not detected by these MAbs, also contains the sequence TTT, suggesting that TTT is not the minimum epitope. Furthermore, the $M2_N$ peptide showed weaker inhibition than the other peptides, suggesting that the epitope for optimum binding may lie in the region PTPTPT common to the $M_{5-19}$ and $M2_C$ peptides. It is of interest that MAb GL-013, raised against gastric carcinoma cells, was shown to react with a TTT-containing minimum epitope on the MUC2 VNTR (Price

**Table III** Expression of the antigens defined by monoclonal antibodies 4F1 and 3A2 in malignant tumours of the colon, stomach, lung, ovary, and breast, and mesotheliomas

| *Tumour type* | *n* | *0* | *Percentage of tumour cells positive*[a] *1–25* | *25–50* | *50–75* | *75–100* |
|---|---|---|---|---|---|---|
| *4F1* | | | | | | |
| Colon | 10 | 1 | 2 | 0 | 4 | 3 |
| Stomach | 10 | 1 | 1 | 0 | 2 | 6 |
| Lung | 10 | 1 | 1 | 2 | 2 | 4 |
| Ovary muc | 7 | 1 | 1 | 1 | 2 | 2 |
| non-muc | 7 | 2 | 2 | 2 | 1 | 0 |
| Breast | 11 | 7 | 1 | 1 | 1 | 1 |
| Mesothelioma | 6 | 1 | 0 | 0 | 0 | 5 |
| *3A2* | | | | | | |
| Colon | 10 | 1 | 5 | 2 | 1 | 1 |
| Stomach | 10 | 3 | 3 | 2 | 2 | 0 |
| Lung | 10 | 4 | 2 | 3 | 0 | 1 |
| Ovary muc | 7 | 3 | 2 | 1 | 1 | 0 |
| non-muc | 7 | 4 | 2 | 1 | 0 | 0 |
| Breast | 11 | 6 | 2 | 2 | 0 | 1 |
| Mesothelioma | 6 | 3 | 0 | 0 | 1 | 2 |

[a]Control mab FM1 was negative on all samples.

*et al.*, 1991), suggesting that 3A2 and 4F1 react with a different part of the MUC2 VNTR. However, the position of an epitope in a synthetic peptide has been shown to affect MAb reactivity (McKenzie & Xing, 1990), and firm conclusions regarding the epitopes for these MAbs cannot be drawn from these experiments. Further experiments with shorter overlapping peptides are needed to define the minimum epitopes of these MAbs.

Differences in the fine specificity of MAbs 3A2 and 4F1 were also illustrated by the reactivity of 3A2 with the $M2_{5-19}$ peptide by inhibition ELISA but not direct ELISA, while 4F1 reacted with this peptide in both assay systems. In addition, the reactivity of 3A2 with the M2 peptide in direct ELISA was dependent on the method of plate coating. This may explain the weaker reactivity of 3A2 with LS174T mucin and CF mucin by direct ELISA. In addition, the consensus sequence of the MUC2 VNTR from intestine is slightly different to that of tracheobronchial tissue (Gerard *et al.*, 1991), so the difference in reactivity with CF mucin may be due to differences in the minimum epitopes of 3A2 and 4F1.

The observations from immunohistochemical staining of both normal and malignant tissues are consistent with 4F1 and 3A2 recognising different epitopes on the protein core of the MUC2 mucin. Cellular distribution of these epitopes in normal gastro-intestinal and respiratory tissues was similar to that described for antigens recognised by polyclonal antibodies prepared against deglycosylated LS174T mucin (Yan *et al.*, 1990) and deglycosylated sputum from a cystic fibrosis patient (Perini *et al.*, 1989). The perinuclear granular staining, seen particularly with 3A2, probably represents detection of the protein core in the endoplasmic reticulum or golgi apparatus prior to the completion of glycosylation. The lack of reactivity of both antibodies with mucin droplets of globlet cells and luminal secretions suggests low reactivity with the mature mucin in normal cells. The observation that not all cells of a given type showed equal expression of either epitope is consistent with the findings of other studies utilising various antibodies to colonic and/or respiratory mucins (Yan *et al.*, 1990; Perini *et al.*, 1989; Podolsky *et al.*, 1986; Finkbeiner & Basbaum, 1988). These findings suggest either phenotypic or stage dependent maturational differences may exist in mucin production patterns of morphologically similar cells.

Detection of the 4F1 and 3A2 epitopes in colonic, gastric, lung, breast, and ovarian cancers demonstrates continued MUC2 production by at least some carcinomas derived from these organs. The expression of both epitopes in some malignancies was much greater than their expression by corresponding non-malignant cells. Increased expression in cancers could reflect either increased production and/or underglycosylation of MUC2. The loss of compartmentalisation of granular cytoplasmic staining is consistent with the loss of polarity that occurs following malignant transformation of epithelial cells. Larger numbers of specimens are required to fully assess the relative expression in different histological types. However, it appears the degree of differentiation has little effect on expression in colon and gastric carcinomas. MUC2 was expressed in both mucinous and non-mucinous ovarian carcinomas. Unfortunately, this finding prevents the use of MUC2 detection for differential diagnosis of gastrointestinal and mucinous ovarian pelvic and peritoneal malignancies. In addition, MUC2 was also expressed by mesotheliomas, and these MAbs could not be used for the differential diagnosis of mesothelioma and adenocarcinoma. The 4F1 and 3A2 epitope expression detected in some cases of breast carcinoma is consistent with the findings of Yan *et al.* (1990) who found a small percentage of cells positive for antibodies against deglycosylated colon mucin in one of five breast cancers, and Jany *et al.* (1991) who demonstrated the presence of MUC2 RNA in mammary tissue. Although antigen was not detected in normal breast duct epithelium, further analysis is required as MUC2 may be expressed by foetal or lactating breast epithelium.

These antibodies should prove useful in the study of MUC2 structure and function, and also have potential as diagnostic and therapeutic agents. The production of second generation anti-peptide antibodies represents a useful method of producing anti-tumour reagents, and this technology may also be applied to the production of MAbs to the other mucin core proteins.

The authors wish to acknowledge Louise Ramm and Sharma Khan for technical assistance, and Drs Gordon Wright and A. Firouz-Abadi for providing pathological material.

### References

AUBERT, J.-P., PORCHET, N., CREPIN, M., DUTERQUE-COQUILLAUD, M., VERGNES, G., MAZZUCA, M., DEBUIRE, B., PETITREZ, D. & DEGAND, P. (1991). Evidence for different human tracheobronchial mucin peptides deduced from nucleotide cDNA sequences. *Am. J. Resp. Cell. Mol. Biol.*, **5**, 178–185.

BHARGAVA, A.K., PETRELLI, N.J., MYERS, M.M., KHAN, S., FITZPATRICK, J., BURKE, P., ANSELMINO, L. & MANDERINO, G. (1989). A new breast cancer marker (BCM) in monitoring disease status. *J. Tumor Marker Oncol.*, **4**, 373–381.

CLARK, G.J., TOBIAS, G.H., PIETERSZ, G.A., CLASSON, B.J., WALKER, I.D., MCKENZIE, I.F.C. & DEACON, L.J. (1988). Isolation of a cDNA clone from the murine CD4 antigen. *Transplant. Proc.*, **20**, 45–48.

DESAI, V.C., NAZIRUDDIN, B., GRAVES, D.C., DE LA ROCHA, S.R. & SACHDEV, G.P. (1991). Production and characterisation of monoclonal antibodies to purified deglycosylated cystic fibrosis respiratory mucin: evidence for the presence of four immunologically distinct epitopes. *Hybridoma*, **10**, 285–296.

DEVINE, P.L., WARREN, J.A., WARD, B.G., MCKENZIE, I.F.C. & LAYTON, G.T. (1990*a*). Glycosylation and the exposure of tumor-associated epitopes on mucins. *J. Tumor Marker Oncol.*, **5**, 11–26.

DEVINE, P.L., WARREN, J.A., CLARK, B.A., LAYTON, G.T., WARD, B.G., MACDONALD, B., XING, P.-X. & MCKENZIE, I.F.C. (1990*b*). The complexity of cancer-associated epitope expression on antigens produced by ovarian tumor cells. *J. Tumor Marker Oncol.*, **5**, 321–339.

DEVINE, P.L., LAYTON, G.T., CLARK, B.A., BIRRELL, G.W., WARD, B.G., XING, P.-X. & MCKENZIE, I.F.C. (1991). Production of MUC1 and MUC2 mucins by human tumor cell lines. *Biochem. Biophys. Res. Commun.*, **178**, 593–599.

DEVINE, P.L. & BIRRELL, G.W. (1992). A method for screening large numbers of samples on immunoblots. *J. Immunol. Meth.*, **149**, 143–144.

DEVINE, P.L. & MCKENZIE, I.F.C. (1992). Mucins: structure, function, and association with malignancy. *BioEssays*, **14**, 619–625.

DEVINE, P.L., BIRRELL, G.W., WHITEHEAD, R.H., HARADA, H., XING, P.-X. & MCKENZIE, I.F.C. (1992). Expression of MUC1 and MUC2 mucins by human tumor cell lines. *Tumor Biol.*, **13**, 267–276.

FINKBEINER, W.E. & BASBAUM, C.B. (1988). Monoclonal antibodies directed against human airway secretions. Localization and characterization of antigens. *Am. J. Pathol.*, **131**, 290–297.

GENDLER, S.J., BURCHELL, J.M., DUHIG, T., LAMPORT, D., WHITE, R., PARKER, M. & TAYLOR-PAPADIMITRIOU, J. (1987). Cloning of partial cDNA encoding differentiation and tumor-associated mucin glycoproteins expressed by human mammary epithelium. *Proc. Natl Acad. Sci. USA*, **84**, 6060–6064.

GENDLER, S., TAYLOR-PAPADIMITRIOU, J., DUHIG, T., ROTHBARD, J. & BURCHELL, J.M. (1988). A highly immunogenic region of a human polymorphic epithelial mucin expressed by carcinomas is made up of tandem repeats. *J. Biol. Chem.*, **263**, 12820–12823.

GERARD, C., EDDY, R.L. & SHOWS, T.B. (1990). The core polypeptide of cystic fibrosis tracheal mucin contains a tandem repeat structure – evidence for a common mucin in airway and gastrointestinal tissue. *J. Clin. Invest.*, **86**, 1921–1927.

GEYSON, H.M., MELOEN, R.H. & BARTELING, S.J. (1984). Use of peptide synthesis to probe viral antigens for epitopes to a resolution of a single amino acid. *Proc. Natl Acad. Sci. USA*, **81**, 3998–4002.

GROSS, E. (1967). The cyanogen bromide reaction. *Meth. Enzymol.*, **11**, 238–255.

GUM, J.R., BYRD, J.C., HICKS, J.W., TORIBARA, N.W., LAMPORT, D.T.A. & KIM, Y.S. (1989). Molecular cloning of human intestinal mucus cDNAs. Sequence analysis and evidence for genetic polymorphism. *J. Biol. Chem.*, **264**, 6480–6487.

GUM, J.R., HICKS, J.W., SWALLOW, D.M., LEGACE, R.L., BYRD, J.C., LAMPORT, D.T.A., SIDDIQUI, B. & KIM, Y.S. (1990). Molecular cloning of cDNAs derived from a novel human intestinal mucin gene. *Biochem. Biophys. Res. Commun.*, **171**, 407–415.

HODGES, R.S. & MERRIFIELD, R.B. (1975). Monitoring a solid phase peptide synthesis by an automated spectrophotometric picrate method. *Anal. Biochem.*, **65**, 241–272.

HOUGHTON, R.A. (1985). General method for the rapid solid phase synthesis of large numbers of peptides: specificity of antigen-antibody interaction at the level of individual amino acids. *Proc. Natl Acad. Sci. USA*, **82**, 5131–5135.

HUMAN GENE MAPPING NOMENCLATURE COMMITTEE (1989). Tenth International Workshop on Human Gene Mapping. *Cytogenet. Cell Genet.*, **51**, 13–66.

JANY, B.H., GALLUP, M.W., YAN, P.-S., GUM, J.R., KIM, Y.S. & BASBAUM, C.B. (1991). Human bronchus and intestine express the same mucin gene. *J. Clin. Invest.*, **87**, 77–82.

LAYTON, G.T., STANWORTH, D.R. & AMOS, H.E. (1987). The specificity of murine polyclonal and monoclonal antibodies to the haptenic drug chlorhexidine induced by chlorine-generated chlorhexidine protein conjugates. *Clin. Exp. Immunol.*, **69**, 157–165.

LAYTON, G.T., DEVINE, P.L., WARREN, J.A., BIRRELL, G., XING, P.-X., WARD, B.G. & MCKENZIE, I.F.C. (1990). Monoclonal antibodies reactive with the breast carcinoma-associated mucin core protein repeat sequence peptide also recognise the ovarian carcinoma-associated sebaceous gland antigen. *Tumor Biol.*, **11**, 274–286.

MCGUCKIN, M.A., OWENS, M., WRIGHT, R.G., MCKENZIE, I.F.C. & WARD, B.G. (1990). Demonstration of seven tumor-associated antigens in epithelial ovarian cancer by immunohistochemistry using monoclonal antibodies. *J. Tumor Marker Oncol.*, **5**, 87–94.

MCKENZIE, I.F.C. & XING, P.-X. (1990). Mucins and breast cancer: recent immunological advances. *Cancer Cells*, **2**, 75–78.

PERINI, J.-M., MARIANNE, T., LAFITTE, J.-J., LAMBLIN, G., ROUSSEL, P. & MAZZUCA, M. (1989). Use of antiserum against deglycosylated human mucins for cellular localization of their peptide precursors: antigenic similarities between bronchial and intestinal mucins. *J. Histochem. Cytochem.*, **37**, 869–875.

PODOLSKY, D.K., FOURNIER, D.A. & LYNCH, K.E. (1986). Human colonic goblet cells. Demontration of distinct subpopulations defined by mucin-specific monoclonal antibodies. *J. Clin. Invest.*, **77**, 1263–1271.

PORCHET, N., VAN CONG, N., DUFFOSSE, J., AUDIE, J.P., GUYONNET-DUPERAT, V., GROSS, M.S., DENIS, C., DEGAND, P.L, BERNHEIM, A. & AUBERT, J.P. (1991). Molecular cloning and chromosomal localisation of a novel human tracheo-bronchial mucin cDNA containing tandemly repeated sequences of 48 base pairs. *Biochem. Biophys. Res. Commun*,. **175**, 414–422.

PRICE, M.R., PUGH, J.A., HUDECZ, F., GRIFFITHS, W., JACOBS, E., SYMONDS, I.M., CLARKE, A.J. & BALDWYN, R.W. (1990). C595 – a monoclonal antibody against the protein core of human urinary epithelial mucin commonly expressed in breast carcinomas. *Br. J. Cancer*, **61**, 681–686.

PRICE, M.R., SEKOWSKI, M., YANG, G.-L., DURRANT, L.G., ROBINS, R.A. & BALDWIN, R.W. (1991). Reactivity of anti-(human gastric carcinoma) monoclonal antibody with core-related peptides of gastrointestinal mucin. *Cancer Immunol. Immunother.*, **33**, 80–84.

SAFI, F., KOHLER, I., ROTTINGER, E. & BEGER, H.-G. (1991). The value of the tumour marker CA15-3 in diagnosing and monitoring breast cancer. *Cancer*, **68**, 574–582.

SHIBIER, O., HAMPTON, S.M. & MARKS, V. (1988). An immunisation protocol which enhances the frequency of antigen-specific monoclonal antibody production. *J. Immunol. Meth.*, **114**, 49–52.

SKERRITT, J.H. & HILL, A.S. (1990). Monoclonal antibody sandwich enzyme immunoassays for determination of gluten in foods. *J. Agric. Food Chem.*, **38**, 1771–1778.

WARD, B.G., MCGUCKIN, M.A., RAMM, L.E., COGLAN, M., SANDERSON, B., TRIPCONY, L. & FREE, K.E. (1993). The management of ovarian carcinoma is improved by the use of cancer-associated serum antigen (CASA) and CA125 assays. *Cancer*, **71**, 430–438.

WHITEHEAD, R.H., MACRAE, F.A., ST. JOHN, J.B. & MA, J. (1985). A colon cancer cell line (LIM1215) derived from a patient with inherited nonpolyposis colorectal cancer. *J. Natl Cancer. Inst.*, **74**, 759–765.

WHITEHEAD, R.H., ZHANG, H.H. & HAYWARD, I.P. (1992). Characterisation of a panel of colon carcinoma cell lines using tissue-specific markers. *Immunol. Cell. Biol.*, **70**, 227–236.

XING, P.-X., TJANDRA, J.J., STACKER, S.A., TEH, J.G., THOMPSON, C.H., MCLAUGHLIN, P.J. & MCKENZIE, I.F.C. (1989). Monoclonal antibodies reactive with mucin expressed in breast cancer. *Immunol. Cell Biol.*, **67**, 183–195.

XING, P.-X., REYNOLDS, K., TJANDRA, J.J., TANG, X.-L. & MCKENZIE, I.F.C. (1990). Synthetic peptides reactive with anti-human milk fat globule monoclonal antibodies. *Cancer Res.*, **50**, 89–96.

XING, P.-X., PRENZOSKA, J., LAYTON, G.T., DEVINE, P.L. & MCKENZIE, I.F.C. (1992). Second generation monoclonal antibodies to intestinal MUC2 peptide reactive with colon cancer. *J. Natl Cancer Inst.*, **84**, 699–703.

YAN, P.-S., HO, S.B., ITZKOWITZ, S.H., BYRD, J.C., SIDDIQUI, B. & KIM, Y.S. (1990). Expression of native and deglycosylated colon cancer mucin antigens in normal and malignant epithelial tissues. *Lab. Invest.*, **62**, 698–706.

ZEGERS, N., GERRITSE, K., DEEN, C., BOERSMA, W. & CLAASSEN, E. (1990). An improved conjugation method for controlled covalent coupling of synthetic peptides to proteins using glutaraldehyde in dialysis method. *J. Immunol. Meth.*, **130**, 195–200.